# *STAT4* (rs7574865) Polymorphism and Serum STAT4 Levels in Rheumatoid Arthritis Susceptibility, Severity, and Treatment Response: A Case-Control Study in Iraqi Patients

**DOI:** 10.30699/ijp.2025.2064449.3485

**Published:** 2025-08-15

**Authors:** Amna R. Abdalhussein, Karrar S. Zayed

**Affiliations:** Department of Laboratory Investigations, Faculty of Science, University of Kufa, Najaf, Iraq

**Keywords:** Rheumatoid arthritis, STAT4, Polymorphism, PCR–RFLP, Methotrexate

## Abstract

**Background & Objective:**

Signal Transducer and Activator of Transcription 4 (STAT4) is a key transcription factor involved in immune signaling pathways and has been implicated in susceptibility to rheumatoid arthritis (RA) across various populations. However, its role in Iraqi Arab cohorts remains largely unexplored.

**Methods:**

A case–control study was conducted including 68 RA patients and 39 age- and sex-matched apparently healthy individuals. The association between the STAT4 (rs7574865) polymorphism, serum STAT4 levels, RA susceptibility, and therapeutic response to methotrexate (MTX), MTX + infliximab, and tofacitinib was investigated using PCR–RFLP and ELISA techniques.

**Results:**

The frequencies of the GT and (GT+TT) genotypes were significantly higher among RA patients than in controls under codominant and dominant models (*P* = .003 and *P* < .001, respectively). The T allele was also significantly more frequent in RA patients than in healthy individuals (*P* = .001). GT and TT genotypes were significantly associated with severe disease (*P* = .042) and poor response to tofacitinib compared with the GG genotype (*P* = .042). Serum STAT4 levels were markedly elevated in RA patients compared with controls (*P* = .01), particularly in those with severe RA (*P* = .042).

**Conclusion:**

** The STAT4 rs7574865 T allele may contribute to increased susceptibility to RA, greater disease severity, and reduced responsiveness to tofacitinib therapy in Iraqi patients.**

## Introduction

Rheumatoid arthritis (RA) is a chronic autoimmune inflammatory disease that primarily affects synovial joints, leading to progressive bone and cartilage destruction, disability, and systemic complications (1). RA is more prevalent in women and typically presents between the fourth and sixth decades of life. Recent global estimates indicate that RA affects approximately 18 million individuals worldwide, with a prevalence of about 0.46%, making it a major cause of disability among middle-aged women (2). The disease imposes a considerable socioeconomic burden and generates substantial healthcare costs.

The etiology of RA is multifactorial, involving complex interactions between genetic and environmental factors (3,4). Among the genetic contributors, the signal transducer and activator of transcription 4 (STAT4) gene has emerged as a key immune regulator. STAT4 mediates interleukin-12 and type I interferon signaling, driving the differentiation of Th1 and Th17 cells. Located on chromosome 2q32.2, STAT4 encodes a transcription factor that regulates several proinflammatory cytokines. The rs7574865 (G>T) polymorphism in intron 3 of STAT4 has been associated with increased susceptibility to several autoimmune diseases, including systemic lupus erythematosus, multiple sclerosis, and RA (5).

Recent studies have shown that the T allele of rs7574865 in STAT4 is strongly linked to increased RA risk, particularly in Asian and Middle Eastern populations (6,7). This polymorphism has also been associated with disease severity (8). However, conflicting evidence exists, as some studies in specific ethnic groups have failed to confirm this association, suggesting that genetic background and environmental exposures may influence its impact (9,10).

Therapeutic management of RA often involves methotrexate, infliximab, and tofacitinib, but treatment outcomes vary across individuals. Genetic factors, including STAT4 polymorphisms, may contribute to these differences. The rs7574865 T allele has been linked to enhanced STAT4 activity, which could affect drug efficacy and side-effect profiles (11,12). Despite these findings, no study has evaluated the association of the STAT4 rs7574865 polymorphism with RA susceptibility, severity, or therapeutic response in Iraqi Arab populations.

Therefore, this study aimed to investigate the correlation between STAT4 rs7574865 polymorphism and serum STAT4 levels with RA susceptibility, disease severity, and treatment response to methotrexate, infliximab, and tofacitinib in Iraqi patients.

## Materials and Methods

### Study Design

Five millilitres peripheral venous blood samples were collected from each of the participants. The samples were divided in two portions: 3 ml were placed in EDTA vials for genomic DNA extraction and stored at −20°C until analysis, following protocols commonly used in genetic studies on *STAT4 *rs7574865 polymorphism (5). The remaining 2 ml were stored at room temperature for 30–60 minutes to allow clotting, then centrifuged to separate serum. Serum was separated and stored at −20°C for later quantification of STAT4 protein levels using enzyme-linked immunosorbent assay (ELISA) (13).

### STAT4 (rs7574865) genotyping

 Genomic DNA from whole blood was extracted using a commercially available DNA Extraction Mini Kit (Qiagen, Hilden, Germany) according to the manufacturer’s instructions. The rs7574865 polymorphism of *STAT4* was detected using polymerase chain reaction followed by restriction fragment length polymorphism (PCR-RFLP). Amplification primers were identical to those used in prior research (5). PCR conditions consisted of an initial denaturation at 95°C for 5 minutes, followed by 35 cycles of 95°C for 30 seconds (denaturation), 58°C for 30 seconds (annealing), and 72°C for 30 seconds (extension), with a final extension at 72°C for 7 minutes, matching protocols in recent RA polymorphism studies. The PCR products were digested using *HpaI* restriction endonuclease (Thermo Scientific, USA). A 147 bp fragment was amplified and subsequently digested. The digestion patterns were as follows: GG genotype (wild-type) showed a single uncut band of 147 bp; GT genotype (heterozygous) displayed two bands 147 bp, and 122 bp; and TT genotype (homozygous mutant) yielded two digested bands of 122 bp and 25 bp—results that are consistent with previous agarose gel-based genotyping reports (14,15). Moreover, the TT genotype, which theoretically produces two fragments of 122 bp and 25 bp, consistently lacked visible detection of the 25 bp band. This absence is likely due to the fragment’s small size and the limitations of 2% agarose gel electrophoresis, where DNA fragments smaller than 100 bp are often difficult to visualize. These findings are consistent with the observations reported by El-Saadany et al. (16). Digested PCR products were resolved on 2.5% agarose gel stained with ethidium bromide and visualized under UV transillumination. Positive and negative controls were included in every experiment to ensure enzymatic efficiency and genotyping reliability.

### Quantification of STAT4 level

 Serum levels of STAT4 were measured according to the manufacturer's protocol using a human-specific ELISA kit (PT Lab, USA). This sandwich-type assay utilized HRP-conjugated antibodies for detection. Serum samples (50 µl each, in duplicates) were added in wells of a pre-coated microtiter plate and incubated at 37°C for 60 minutes with continues shaking at 250 rpm. After washing, chromogenic substrate was added and incubated in the dark for 15 minutes. The reaction was stopped with 50 µl of stop solution, and absorbance was read at 450 nm using a microplate ELISA reader (17). STAT4 levels were quantified based on a standard curve run against known concentrations, consistent with methodologies applied in previous studies examining STAT4 levels in autoimmune diseases (8).

### Treatment Response Criteria

#### Methotrexate (MTX)

 The therapeutic response to methotrexate (MTX) monotherapy was evaluated based on the criteria established by the American College of Rheumatology (ACR), which serve as the gold standard for assessing clinical improvement in rheumatoid arthritis. These criteria quantify the percentage of improvement across key domains of disease activity as follows:

**ACR20**: Indicates a minimum of 20% improvement in both tender and swollen joint counts, along with a 20% improvement in at least three of the following domains: patient global assessment, physician global assessment, pain scale, functional ability (e.g., Health Assessment Questionnaire [HAQ]), and acute-phase reactants (either ESR or CRP).

**ACR50** and **ACR70**: Reflect 50% and 70% improvements, respectively, across the same clinical parameters (18).

### MTX Combined with Infliximab

 For patients receiving combination therapy with MTX and infliximab (a TNF-α inhibitor), the ACR20/50/70 criteria were similarly employed to evaluate treatment response. In addition, the **Disease Activity Score for 28 joints (DAS28)** was used for a more nuanced assessment, particularly in the context of biologic therapy. The DAS28 incorporates tender and swollen joint counts (out of 28 joints), the patient's global health assessment, and a measure of systemic inflammation (ESR or CRP). DAS28-based responses are categorized as follows:

**EULAR Good Response**: A reduction in DAS28 >1.2 points with a final score ≤3.2.

**EULAR Moderate Response**: A reduction between 0.6 and 1.2 points.

**Remission**: Achieved with a DAS28 score <2.6 (19).

**Tofacitinib**

 Tofacitinib, a Janus kinase (JAK) inhibitor and a member of the targeted synthetic DMARD (tsDMARD) class, was evaluated using both the ACR20/50/70 criteria and DAS28 scoring. These standardized assessment tools enable direct comparison of tofacitinib’s efficacy with other therapeutic regimens for RA, facilitating a uniform approach to measuring clinical outcomes (20).

### Sample Size Justification

 The sample size for this study was determined based on power analysis to ensure sufficient statistical strength to meet the primary research objectives. An a priori calculation using G Power software guided recruitment efforts.

For serum STAT4 level comparison (continuous variable)

 Assuming a significance level (α) of 0.05, a statistical power of 80% (0.80), and a medium-to-large effect size (Cohen's d = 0.6), the required sample size was estimated at approximately 45 participants per group.

For genetic association analysis of the STAT4 rs7574865 polymorphism (categorical variable)

 Under the assumptions of α = 0.05, power = 0.80, an expected odds ratio (OR) of 2.5, and a minor allele frequency (MAF) of 0.25 in the control group, the estimated requirement was around 60 cases and 60 controls. Ultimately, the study enrolled 68 patients with rheumatoid arthritis and 39 apparently healthy controls, totaling 107 participants. While this deviated slightly from the initial estimates due to logistical limitations during recruitment, post hoc power analyses confirmed the adequacy of the final sample. Specifically:

Power for STAT4 serum level comparison was calculated at 0.91 (91%).

Power for genetic association analysis reached 0.81 (81%) for detecting an OR of 2.5.

 Both values exceed the conventional threshold of 80%, confirming that the sample size was sufficient to detect clinically relevant differences with high statistical reliability.

### Statistical analysis

Statistical analyses were performed using the Statistical Package for the Social Sciences (SPSS), version 23.0 (IBM, USA). Continuous variables are presented as mean ± standard deviation (SD). The chi-square test was employed to compare allele and genotype frequencies. Differences among multiple groups were evaluated using one-way analysis of variance (ANOVA), followed by Tukey’s post hoc test for pairwise comparisons. Odds ratios (ORs) and 95% confidence intervals (CIs) were calculated to assess the association between rheumatoid arthritis risk and specific genotypes. A *p*-value less than 0.05 was considered statistically significant.

**Fig. 1:**
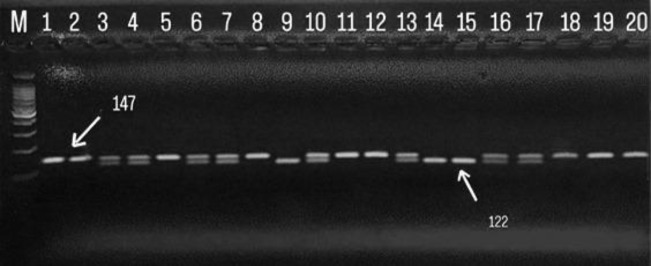
illustrates the genotyping of the *STAT4* polymorphism (rs7574865) using RFLP-PCR with 2% agarose gel electrophoresis and the *HpaI* enzyme. The PCR product (147 bp) was digested by *HpaI*. Band M displays the GeneRuler™ 100 bp DNA Ladder, serving as a molecular weight marker. The homozygous wild-type genotype (GG) is represented by a single undigested band at 147 bp, as seen in bands 1, 2, 5, 8, 11, 12, 18, 19, and 20. The heterozygous genotype (GT) is indicated by two fragments, at 147 bp and 122 bp, found in bands 3, 4, 6, 7, 10, 13, 16, and 17. For the TT genotype, while 122 bp and 25 bp bands were expected, the 25 bp band remained undetected due to its small size. Therefore, only the 122 bp band was clearly visible for this genotype.

## Results

The genotype and allele frequency of *STAT4 *(rs7574865) polymorphism have been determined, as listed in figure 1 and table 1.

 The GT genotype in codominant model was significantly more frequent among RA patients compared to apparently healthy controls (48.5% vs. 20.5%), (*p*=0.003). In the dominant model (GT + TT vs. GG), this association was even more pronounced (*p*=0.0005) in RA patients than control, with an OR of 5.32 and 95% CI ranging from 2.24 to 12.61. In contrast, the recessive model (TT vs. GG + GT) revealed no statistically significant difference between RA patients and healthy individuals (*p*=0.610). Furthermore, the frequency of the T allele was significantly higher among RA patients (43.4%) compared to controls (17.9%) (*p*=0.001; OR = 3.5, 95% CI: 1.79–6.84) as presented in table 1.

Furthermore, a significant association was also observed between *STAT4 *(rs7574865) genotypes and disease severity among RA patients, as assessed by the DAS28-CRP index, carriers of the TT genotype exhibited the highest proportion of severe disease activity (35.3%) whereas the GG and GT genotypes were more frequently observed among patients with mild (42.9%) and moderate (51.9%) activity (*p*=0.042). In contrast, DAS28-ESR and CADI severity indices parameters showed no significant association between any of the three genotypes and disease severity (*p*= 0.061 and 0.276) respectively as illustrated in table 2. 

**Table 1:** *STAT4* (rs7574865) genotypes and allele frequencies in RA patients and healthy apparently

Models Genotype			Group		Total	Odd ratio 95% CI	* p. value	
			Healthy apparently	RA				
Codominant GG GT TT		N	28	22	50	1 Reference	-	
		%	71.8%	32.4%	46.7%			
		N	8	33	41	5.25 (2.02-13.61)	0.003	
		%	20.5%	48.5%	38.3%			
		N	3	13	16	5.51 (1.39-21.79)	0.07	
		%	7.7%	19.1%	15.0%			
Total		N	39	68	107			
		%	100.0%	100.0%	100.0%			
Dominant GG GT + TT		N	28	22	50	1 Reference	-	
		%	71.8%	32.3%	46.7%	5.32 (2.24-12.61)	0.0005	
		N	11	46	57			
		%	28.2%	67.6%	53.3%			
Total		N	39	68	107			
		%	100%	100%	100%			
Recessive GG+GT TT		N	36	55	91	1 Reference		-
		%	92.3%	80.9%	85.1%			
		N	3	13	16	2.83 (0.75-10.65)		0.610
		%	7.7%	19.1%	14.9%			
Total		N	39	68	107			
		%	100%	100%	100%			
Allele frequency	G T	N	64	77	141	1 (Reference)		-
		%	82.1%	56.6%	65.9%			
		N	14	59	73	3.5 (1.79-6.84)		0.001
		%	17.9%	43.4%	34.1%			
Total		N	78	136	214			
		%	100%	100%	100%			

*p. values were corrected by multiple comparisons using the Bonferroni method

**Table 2:** Association of* STAT4 *(rs7574865) genotypes and disease severity parameters in RA patients

RA severity parameter			GG	GT	TT	Total	* *p*. value
DAS 28-CRP	Mild	N	3	4	0	7	0.042
		%	42.9%	57.1%	0.0%	100.0%	
	Moderate	N	12	14	1	27	
		%	44.4%	51.9%	3.7%	100.0%	
	Severe	N	7	15	12	34	
		%	20.6%	44.1%	35.3%	100.0%	
Total		N	22	33	13	68	
		%	32.4%	48.5%	19.1%	100.0%	
DAS 28- ESR	Mild	N	4	4	0	8	0.061
		%	50.0%	50.0%	0.0%	100.0%	
	Moderate	N	10	18	2	30	
		%	33.3%	60.0%	6.7%	100.0%	
	Severe	N	8	11	11	30	
		%	26.7%	36.7%	36.7%	100.0%	
Total		N	22	33	13	68	
		%	32.4%	48.5%	19.1%	100.0%	
CDAI	Mild	N	6	5	1	12	0.276
		%	50.0%	41.7%	8.3%	100.0%	
	Moderate	N	8	9	1	18	
		%	44.4%	50.0%	5.6%	100.0%	
	Severe	N	8	19	11	38	
		%	21.1%	50.0%	28.9%	100.0%	
Total		N	22	33	13	68	
		%	32.4%	48.5%	19.1%	100.0%	

*p. values were corrected by multiple comparisons using the Bonferroni method

**Table 3:** Association of *STAT4* (rs7574865) genotypes and RA response to (MTX, MTX +Infliximab, Tofacitinib) treatments

Treatment			GG	GT	TT	Total	* *p*. value
MTX	Yes	N	10	5	0	15	0.107
		%	66.7%	33.3%	0.0%	100.0%	
	No	N	1	2	2	5	
		%	20.0%	40.0%	40.0%	100.0%	
Total		N	6	12	2	20	
		%	30.0%	60.0%	10.0%	100.0%	
MTX +Infliximab	Yes	N	8	7	1	16	0.06
		%	50.0%	43.8%	6.3%	100.0%	
	No	N	1	5	6	12	
		%	8.3%	41.7%	50.0%	100.0%	
Total		N	9	13	6	28	
		%	32.1%	46.4%	21.4%	100.0%	
Tofacitinib	Yes	N	7	5	0	12	0.042
		%	58.3%	41.7%	0.0%	100.0%	
	No	N	1	3	4	8	
		%	12.5%	37.5%	50.0%	100.0%	
Total		N	8	8	4	20	
		%	40.0%	40.0%	20.0%	100.0%	

*p. values were corrected by multiple comparisons using the Bonferroni method

Furthermore, analysis of the association between *STAT4 *(rs7574865) genotypes and treatment response revealed that only the tofacitinib group demonstrated that the TT genotype was consistently linked to poor therapeutic outcomes (0%) while GG genotype was demonstrated a good response to and tofacitinib (58.3%) (*p* =0.042), while associations with MTX (*p*=0.107) and MTX+ Infliximab (p=0.06) were not significantly as illustrated in table 3. 

Moreover, serum STAT4 level was significantly elevated in RA patients (0.37 ± 0.24 ng/mL) compared to apparently healthy individuals (0.25 ± 0.16 ng/mL), with a statistically significant difference (*p* = 0.010) as shown in table 4.

Moreover, there was no statistically significant difference in serum STAT4 levels among RA patients treated with MTX, MTX +Infliximab and Tofacitinib (*p*=0.135) as shown in table 5.

**Table 4:** Serum STAT4 level in Apparently healthy and RA patients

	group	N	Mean	+SD	*p*. value
STAT4	Apparently healthy	39	0.25	0.16	0.010

**Table 5:** Serum STAT4 level in in RA treated with MTX, MTX +Infliximab and Tofacitinib drugs

Treatment	N	Mean	+SD	95% CI		*p. value
				Lower Bound	Upper Bound	
MTX	20	0.46	0.18	0.38	0.55	0.135
MTX +Infliximab	28	0.39	0.26	0.29	0.50	
Tofacitinib	20	0.29	0.17	0.20	0.37	

*p. values were corrected by multiple comparisons using the Bonferroni method

**Table 6:** Association of STAT4 and disease severity parameters in RA patients

		N	Mean	+SD	95% CI		p. value	* *p*. value
					Lower Bound	Upper Bound		
DAS 28-CRP	Mild	7	0.25	0.11	0.14	0.35	0.006	0.018
	Moderate	27	0.29	0.15	0.23	0.35		
	Severe	34	0.43	0.22	0.35	0.51		
DAS 28-ESR	Mild	8	0.27	0.09	0.19	0.35	0.182	0.546
	Moderate	30	0.33	0.21	0.25	0.41		
	Severe	30	0.40	0.21	0.32	0.48		
CDAI	Mild	12	0.34	0.22	0.27	0.4227	0.857	1.00
	Moderate	18	0.36	0.19	0.24	0.4881		
	Severe	38	0.38	0.17	0.29	0.4689		

*p. values were corrected by multiple comparisons using the Bonferroni method

On the other hand, a significant elevation in serum STAT4 levels was also observed among RA patients with severe disease activity compared to those with mild and moderate activity, as determined by the DAS28-CRP index (p = 0.018). In contrast, no statistically significant differences in STAT4 levels were found when disease activity was assessed using DAS28-ESR (*p* = 0.546) or CDAI (*p* = 1.00) (see table 6). 

Furthermore, no significant differences in STAT4 levels were observed among the three genotypes (GG, GT, TT) (*p* = 0.075), as shown in table 7.

**Table 7:** Association of *STAT4* (rs7574865) genotypes with serum level of STAT4 in RA patients

Genotype	N	Mean	+ SD	95% CI		* *p*. value
				Lower Bound	Upper Bound	
GG	22	0.29	0.16	0.21	0.36	0.075
GT	33	0.36	0.17	0.31	0.42	
TT	13	0.48	0.31	0.29	0.67	

*p. values were corrected by multiple comparisons using the Bonferroni method

## Discussion

This study revealed a statistically significant association between the *STAT4 *(rs7574865) polymorphism and susceptibility to RA in Iraqi patients. Both GT genotype and T allele were significantly overrepresented in the patient group compared to apparently healthy individuals. These findings suggest that the presence of the T allele may increase genetic susceptibility to RA in this population, aligning with previous studies conducted in Asian and Middle Eastern cohorts, where the *STAT4 *(rs7574865) variant was consistently associated with RA risk (10,21). This supports the hypothesis that the STAT4 T allele may serve as a heritable marker contributing to immune dysregulation and chronic inflammation in RA (22). while the dominant model (GT+TT vs. GG) in this study was strongly associated with RA susceptibility, the recessive model (TT vs. GG+GT) did not yield significant results, suggesting that the presence of a single T allele is sufficient to elevate disease risk, while homozygosity may not confer additional susceptibility (23). This pattern aligns with the concept of partial dominance, where the risk allele acts in a dose-independent manner. However, previous findings have shown variability across populations. For instance, a genome-wide association study in a Colombian and European cohort found inconsistent associations between *STAT4 *(rs7574865) and RA, particularly when considering the influence of HLA-DRB1 alleles and other genetic modifiers (9,24). These discrepancies highlight the complex polygenic architecture of RA and emphasize the importance of population-specific genetic screening. Taken together, the results of the current study reinforce the potential of *STAT4* as a susceptibility marker for RA in Arab populations, while also pointing to the need for broader, multiethnic replication studies to better understand its universal and population-specific effects.

 Furthermore, the association between *STAT4* (rs7574865) genotypes and disease severity was statistically significant based on DAS28-CRP. Patients carrying the TT genotype exhibited the highest frequency of severe disease activity, particularly under DAS28-CRP, while GT and GG genotypes were predominantly found among patients with mild to moderate activity levels. These findings underscore a potential contribution of the T allele to disease exacerbation in RA. This is in line with previous findings that demonstrated T allele carriers displayed higher expression of inflammatory mediators and had more aggressive disease progression (25). When evaluating treatment effectiveness, we found a difference in statistical significance between the CDAI and DAS28-CRP scores. While DAS28-CRP showed a significant change, CDAI did not. This difference is likely due to how each score is calculated. CDAI is based only on clinical observations and does not include lab markers, which may make it less sensitive to subtle inflammatory changes. In contrast, DAS28-CRP includes (CRP), a more responsive indicator of inflammation. Additionally, our sample size was likely adequate to detect changes measured by DAS28-CRP, but may not have been large enough to detect smaller or more variable changes captured by CDAI (26). Likewise, this study showed in the Tofacitinib-treated group, TT genotype patients exhibited the poorest therapeutic outcomes, suggesting that kinase inhibitors may have limited effectiveness in genetically predisposed inflammatory states. These patterns are consistent with prior observations reporting diminished response to biologics among carriers of high-risk *STAT4* alleles (7,27). In the Iraqi population, it’s important to take into account environmental and socioeconomic factors that may affect how the STAT4 (rs7574865) gene is expressed and how rheumatoid arthritis (RA) presents or responds to treatment. Long-term exposure to heavy metals like cadmium and lead—common in industrial areas and regions affected by war—can increase oxidative stress and activate inflammatory processes involved in autoimmune diseases. At the same time, nutritional deficiencies, especially in key antioxidants such as selenium, vitamin D, and zinc, are widespread due to economic challenges and limited diets. These deficiencies may contribute to immune system imbalance and higher RA disease activity. On top of that, many patients face poor access to healthcare, delays in diagnosis, and ongoing stress related to conflict, all of which can worsen RA symptoms and reduce the effectiveness of treatments. These combined factors might help explain why the T allele of STAT4 is so strongly linked to higher RA risk and more severe disease. Considering how genes interact with the environment is essential when studying autoimmune conditions in regions with unique environmental and social challenges like Iraq (28).

 Moreover, in terms of protein expression, serum STAT4 levels were significantly elevated in RA patients compared to apparently healthy controls, supporting the role of STAT4-driven transcriptional upregulation in disease progression. The consistent elevation of STAT4 expression in RA patients, as shown across multiple studies, highlights its pivotal role in driving autoimmune inflammation. STAT4 is a transcription factor activated primarily by IL-12 and IL-23 signaling, which are key cytokines in Th1 and Th17 differentiation, respectively. The upregulation of STAT4 leads to increased production of pro-inflammatory cytokines such as IFN-γ and IL-17, both of which are heavily involved in synovial inflammation and joint destruction in RA. The elevated serum levels of STAT4 observed in RA patients (29), and corroborated by subsequent studies (30,31), suggest that STAT4 is not only active at the site of inflammation (i.e., synovium) but also systemically detectable, making it a promising biomarker for disease activity. Furthermore, its correlation with disease severity (as reflected in DAS28 scores) implies that STAT4 might contribute to disease progression and not just its onset. On the other hand, patients with severe RA activity, as measured by DAS28-CRP, displayed significantly higher serum concentrations of STAT4 compared to those with mild or moderate disease. This observation supports previous findings that linked STAT4 expression with increased disease burden in RA (32,33). 

 Despite the strength of our findings, there are some important limitations to consider. First, our sample size was relatively small—especially within the treatment groups divided by genotype-which may have reduced the statistical power and limited how well our results can be applied to the wider population. This may have caused us to miss subtle associations or led to a higher chance of Type II errors. Second, we used the PCR-RFLP method for genotyping. While this technique is widely used and affordable, it might not be sensitive enough to detect very small DNA fragments-like the 25 base pair band expected in the TT genotype. This raises the risk of misclassifying some genotypes, particularly if the fragment is too small to be seen using standard agarose gel electrophoresis. To improve accuracy, future studies should consider using more advanced methods like direct DNA sequencing. Finally, although we applied Bonferroni correction to account for multiple testing and reduce the risk of false positives (Type I errors), this very conservative method might have made it harder to detect true associations. Overall, these technical and statistical limitations highlight the need for larger, multi-center studies to confirm and build on our results.

## Conclusion

This study highlights a potential association between the *STAT4* rs7574865 T allele and increased susceptibility and severity of rheumatoid arthritis in Iraqi patients. The TT genotype was linked to elevated STAT4 serum levels, more severe clinical presentation, and poorer response to methotrexate, infliximab, and tucatinib. While these results are promising, STAT4 genotyping should be considered an investigational biomarker at this stage, pending further validation in larger, ethnically diverse populations to confirm its predictive value and clinical relevance.

## Data Availability

There is no additional data separate from available in cited references.
